# Summer Diarrhœa or Summer Complaint

**Published:** 1886-09

**Authors:** V. D. Lockhart

**Affiliations:** Homer, Ga.


### SUMMER DIARRHŒA OR SUMMER COMPLAINT.

BY V. D. LOCKHART, M. D., HOMER, GA.

This is one of the most common, and, I may say, one of the most important, diseases we are called upon to treat during the summer months, and while I shall not attempt to give your readers anything specially new in the way of treatment, I am prompted to write this article, hoping it may be the means of curing some of the reckless and careless prescribing done by some physicians.

Each case should be carefully examined in reference to the following points: 1st. The character and frequency of the discharges. 2nd. The condition of the gums if the child is teething. 3rd. The kind of food the child is living on; and 4th. The amount of fever present, if any. A careful diagnosis, in other words, is absolutely necessary in each case before commencing treatment. Over-feeding and improper food is one of the most common causes of diarrhœa and dysentery among infants and small children. In the country the mothers of young children are often at work in the hot sun or around a hot stove cooking for the family, which often causes derangement of the system and vitiates the milk. A large majority of these little patients will recover with the simplest kind of medication if the cause be carefully sought out and remedied.

There is no danger from a simple diarrhœa if arrested in time to prevent the disease from assuming a chronic form, or developing into a catarrhal inflammation of the intestinal canal. This brings us to the treatment, and I shall confine myself to the discussion of those methods most useful to the country doctor, for it is useless to talk or write to him about expensive pharmaceutical preparations, for our patients cannot afford them.

In a large majority of cases it is important in the beginning of treatment to clear out the *primæ viæ* and stimulate the liver to normal action. For this purpose nothing is better than small and frequently repeated doses of saccharated calomel, or calomel and Dover's powder, or mercury and chalk with minute doses of ipecac. The aromatic syrup of rhubarb, or appropriate doses of powdered rhubarb combined with bicarb. soda, is also excellent for this purpose.

To control the bowels and prevent the frequent discharges that so much weaken the system, there are a number of mixtures more or less effective. These mixtures may be combined with paregoric or other opiates, but I always prescribe opiates separately, in order that they may not be adminstered too frequently or continued longer than necessary. Bromide of potassium serves an excellent purpose in allaying nervous irritation in those cases complicated with the eruption of the teeth.

The following prescription is very simple and effective:

℞. Bismuthi Subnitrat........................ʒij

Acid. Carbol........................gr.ij

Mucil. Acaciæ........................℥j

Mistura Cretæ........................℥iij

Flavor with a few drops of essence of peppermint, and direct the bottle to be well shaken before each dose, and give teaspoonful every three to six hours. This may be combined with tinct. catechu or other appropriate astringents or opiates as occasion may require.

We are often called to prescribe for patients where there is considerable tenderness of the abdomen, fever, greenish or slimy stools, streaked with blood, tenesmus and other symptoms indicating more or less inflammation, which has occurred rather suddenly, or developed out of a simple diarrhœa. Great relief is afforded by placing the child in a warm bath for a few minutes and afterwards applying a warm poultice of peach leaves sprinkled with laudanum. Here our best and most careful judgment should be exercised, for if the constitution and previous health be impaired, we may expect an unfavorable termination. Generally a dose of castor oil with laudanum should be administered, and after the bowels are cleared out, rest and quiet should be maintained by the continued administration of opiates and laxatives as may be required.

I think Dover's powder is the most useful opiate, and syrup of rhubarb, or rhubarb and bicarb. soda, the most suitable laxatives. Subnitrate of bismuth should be given in combination with the Dover's powder all the while, or alone if the opiate be suspended.

As the disease subsides recourse must be had to tonics and stimulants to restore the strength and appetite.

In conclusion, I will add that we should be more careful to warn mothers to avoid giving young children starchy and saccharine food. I have known children killed outright with the "sugar teat." This is a villainous compound of sugar, butter, biscuit, fruits and the Lord only knows what all. It is placed in the mouths of new-born infants, and they are kept sucking away on this indigestible mass until the digestion is so much impaired that it is a wonder any of them ever live. Where it is necessary to raise a babe by hand, we ought to give the proper directions and see that they are carried out as far as we can.

